# Supplementary material for: Chromothripsis during telomere crisis is independent of NHEJ, and consistent with a replicative origin
Source: Genome Res. 2019 May;29(5):737–49. doi: 10.1101/gr.240705.118 (PMC6499312; doi:10.1101/gr.240705.118)
Supplement: Supplemental Material [file supp_gr.240705.118_Supplemental_file_1.zip › contigs/annotated_contigs/DB96/contig.3.DB96_length_359_mean_cov_6.85793871866.docx]

**DB96_length_359_mean_cov_6.85793871866**

```
GCCATGAAAGCAGAAGGCCGGGGTGGCGGGGGGGGAGGATTGGGAGCCCCGGCCCACAAGCCG|GGGCAACGGGCGAAGACCCCGTC|A
                                                                >chr1:9471744-9471794 -
AAAAAAAAAAAAAAAAAAAAAAAAAA|AATTAGCCAGGCATGGTGGCACACACCTGTAGAAACTACTCAGAAGGCTGAGGTGGGAGAAT
 E=1e-06 p=0e+00 >chr13:98252511-98252668 + E=4e-83
CACTTGAGCCCTGGAGGCAGAGGTTTCTATGAGCCAAGACTGCACCACTGCACTCCACCCTGGGAAAC|GGGCGGCAGCCATCACTACA
                                                                      >chr2:142418009-14
GCTCCAGTCTGGCAGTTCCCCCCCGCTGGTGCCGCGGAAACTGGGCAGTTTGGACCCAGGAAGAATTCCCCAGGGTGTACCACAGCAGC
2418124 + E=9e-58
TGTGGC|N
```
